# MRI Findings of Early-Stage Hyperacute Hemorrhage Causing Extramedullary Compression of the Cervical Spinal Cord in a Dog with Suspected Steroid-Responsive Meningitis-Arteritis

**DOI:** 10.3389/fvets.2017.00161

**Published:** 2017-09-27

**Authors:** Adriano Wang-Leandro, Enrice-Ina Huenerfauth, Katharina Heissl, Andrea Tipold

**Affiliations:** ^1^Department of Small Animal Medicine and Surgery, University of Veterinary Medicine Hannover, Foundation, Hannover, Germany

**Keywords:** magnetic resonance imaging, steroid-responsive meningitis-arteritis, canine, tetraparesis, signal intensity, subarachnoid hematoma, bleeding, immune-mediated vasculitis

## Abstract

A 9-month-old female Weimaraner was presented to the emergency service due to episodes of fever and neck pain. Physical examination revealed a stiff neck posture and elevated body temperature. Shortly after clinical examination was performed, the dog developed peracute onset of non-ambulatory tetraparesis compatible with a C1–C5 spinal cord (SC) lesion. Immediately thereafter (<1 h), MRI of the cervical SC was performed with a 3-T scanner. A left ventrolateral intradural-extramedullary SC compression caused by a round-shaped structure at the level of C3––C4 was evidenced. The structure was iso- to slightly hyperintense in T1-weighted (T1W) sequences compared to SC parenchyma and hyperintense in T2-weighted, gradient echo, and fluid-attenuated inversion recovery. Moreover, the structure showed a strong homogeneous contrast uptake in T1W sequences. Cerebrospinal fluid (CSF) analysis revealed a mixed pleocytosis, as well as elevated protein and erythrocyte count. Early-stage hyperacute extramedullary hemorrhage was suspected due to immune mediated vasculitis. The dog was maintained under general anesthesia and artificial ventilation for 24 h and long-term therapy with corticosteroids and physiotherapy was initiated. Eight weeks after initial presentation, the dog was ambulatory, slightly tetraparetic. Follow-up MRI showed a regression of the round-shaped structure and pleocytosis was not evident in CSF analysis. This report describes an early-stage hyperacute extramedullary hemorrhage, a condition rarely recorded in dogs even in experimental settings.

## Case Description

A 9-month-old female Weimaraner (32.2 kg) was referred to the Department of Small Animal Medicine and Surgery of the University of Veterinary Medicine Hannover because of acute presentation of cervical pain and fever. The dog had no previous history of illness.

The symptoms were first noted by the owners 2 days before presentation when the dog was obtunded, inappetent, unwilling to stand up, and showed a stiff gait. The primary care veterinarian administered Metamizol (50 mg/kg TID, rectally) to reduce the fever.

At admission to the clinic, physical examination revealed a kyphotic posture with stiffness of the neck and head, body temperature of 39.3°C and a marked cervical hyperesthesia. The complete blood count (CBC) showed a leukocytosis of 35.12 × 10^3^ cells/μl [reference interval (RI) 6–12 × 10^3^ cells/μl] with a neutrophilia of 25.29 cells/μl (RI 3–10 × 10^3^ cells/μl). Serum biochemistry revealed a mild elevation of the alkaline phosphatase and lactate values. Furthermore, prothrombin and partial thromboplastin time were elevated (laboratory findings are available in the Table S1 in Supplementary Material).

Approximately 45 min after the general physical examination was performed, the dog’s clinical status worsened acutely. At this time point, besides the cervical hyperesthesia, neurological examination revealed a non-ambulatory tetraparesis with less voluntary movement present on the left limbs. Moreover, all limbs were spastic, spinal reflexes were normal and evaluation of cranial nerves was unremarkable. Therefore, the neuroanatomical localization of the lesion was set within C1–C5 spinal cord (SC) segments.

Immediately thereafter, general anesthesia was induced and MRI of the cervical SC was performed. For image acquisition, the dog was positioned in dorsal recumbency and a 15-channel sensitivity-encoding (SENSE) spine coil was used. Sagittal T1- and T2-weighted (T2W) sequences were performed (TR 11.3, TE 5.2; TR 3,100, TE 120, respectively), as well as transversal T1-weighted (T1W) (TR 11.3, TE 5.2) and T2W (TR 8,000, TE 120), T2*gradient echo (GRE; TR 660, TE 6.9), spectral attenuated inversion recovery (SPAIR; TR 5237, TE 100), and fluid-attenuated inversion recovery (FLAIR; TR 10,000, TE 140) sequences were acquired with a 3-T MRI scanner (Philips Achieva, Eindhoven, The Netherlands). Gadolinium was administered as contrast medium (Dotarem^®^ 0.2 mmol/kg; i.v.) and sagittal and transversal T1W images were acquired post-contrast. Images were analyzed with commercially available software (RadiAnt DICOM Viewer, Version 4.0.3, Poznan, Poland).

**Figure 1:**
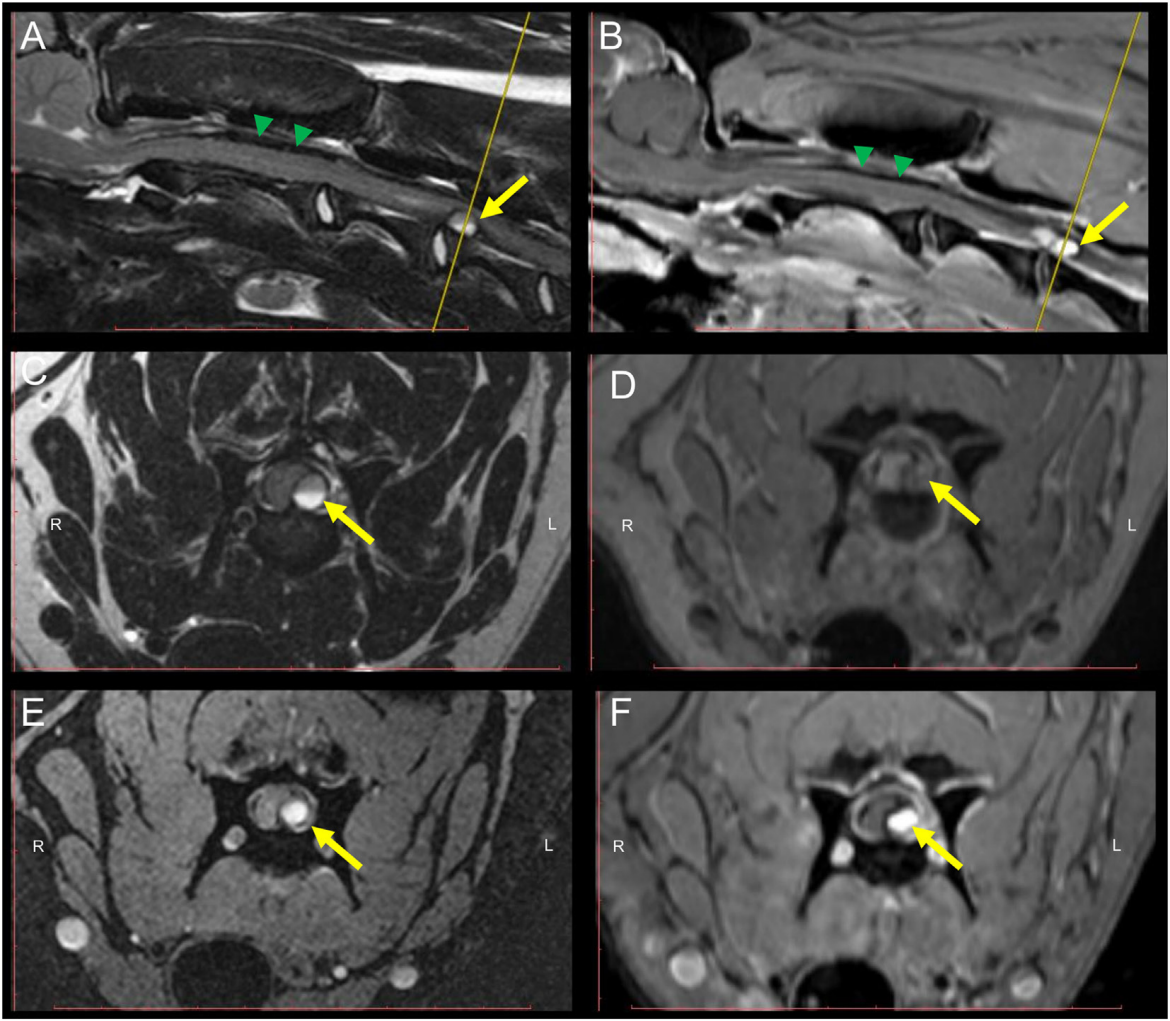
MRI of the cervical spinal cord (SC) of a 9-month-old Weimaraner with peracute onset of non-ambulatory tetraparesis. Sagittal T2W **(A)** and post-contrast T1W **(B)** sequences depict an intradural-extramedullary left sided ventrolateral compression of the SC at the level of C3–C4. The yellow arrow points at the round-shaped structure causing the SC compression. Green arrowheads point to the T2W and T1W inhomogeneous, mostly hypointense material present within the subarachnoidal space dorsal to the SC. Transversal T2W **(C)**, pre-contrast T1W **(D)**, GRE **(E)**, and post-contrast T1W **(F)** sequences. A hyperintense signal of the core of the mass lesion compressing the SC can be evidenced in all sequences. A horizontal fluid-fluid level is noticed within the core of the round-shaped lesion in T2W sequences **(A,C)**. The yellow line in the sagittal T2W and post-contrast T1W **(A,B)** indicates the level at which transversal sequences are depicted. Abbreviations: T2W, T2-weighted; T1W, T1-weighted; GRE, T2*, gradient echo.

MRI revealed an intradural-extramedullary compression of the SC at the level of C3–C4. The compression was caused by a round-shaped structure which showed a high intensity signal in T2W, GRE, SPAIR, and FLAIR sequences and an inhomogeneous iso- to slightly hyperintense signal in T1W sequences (Figure 1). A homogeneous strong contrast enhancement was noticed in T1W sequences after contrast medium administration. Additionally, a well-defined horizontal line dividing the mass into two different signal intensities was evidenced in both acquired planes of T2W sequences.

Furthermore, subarachnoidal space depicted ventral and dorsal to the SC in sagittal views did not display a strong hyperintense signal in T2W images, as characteristic in normal conditions. Instead, an inhomogeneous, mostly hypointense (T2W, T1W, and GRE) signal was present.

After performing a lumbar tap, cerebrospinal fluid (CSF) had a xanthochromic macroscopic appearance and the analysis evidenced a neutrophilic–monocytic mixed pleocytosis (value: 300 cells/μl; RI < 5 cells/μl) with elevated protein content (value: 851.82 mg/dl; RI < 40 mg/dl) and erythrocytes (value: 5,120 cells/μl). IgA levels in serum and CSF were within the upper reference value or elevated, respectively (IgA value for serum: 97.6 µg/ml; RI in serum: <100 μg/ml; IgA value in CSF: 16.4 µg/ml; RI in CSF: <0.2 μg/ml).

Due to the signalement, history, clinical presentation, and findings collected from diagnostic tests, an early-stage hyperacute, intradural-extramedullary compressive hemorrhage of the cervical SC secondary to an immune mediated vasculitis was suspected. A long-term protocol starting with high dose glucocorticosteroids was applied as previously reported (1), and artificial ventilation was administered for the first 24 h. During hospitalization, the dog developed a urinary tract infection which was treated with antibiotics according to antibiogram results and received daily physiotherapy sessions. The patient showed clinical improvement and was discharged from the clinic 16 days after initial presentation.

**Figure 2:**
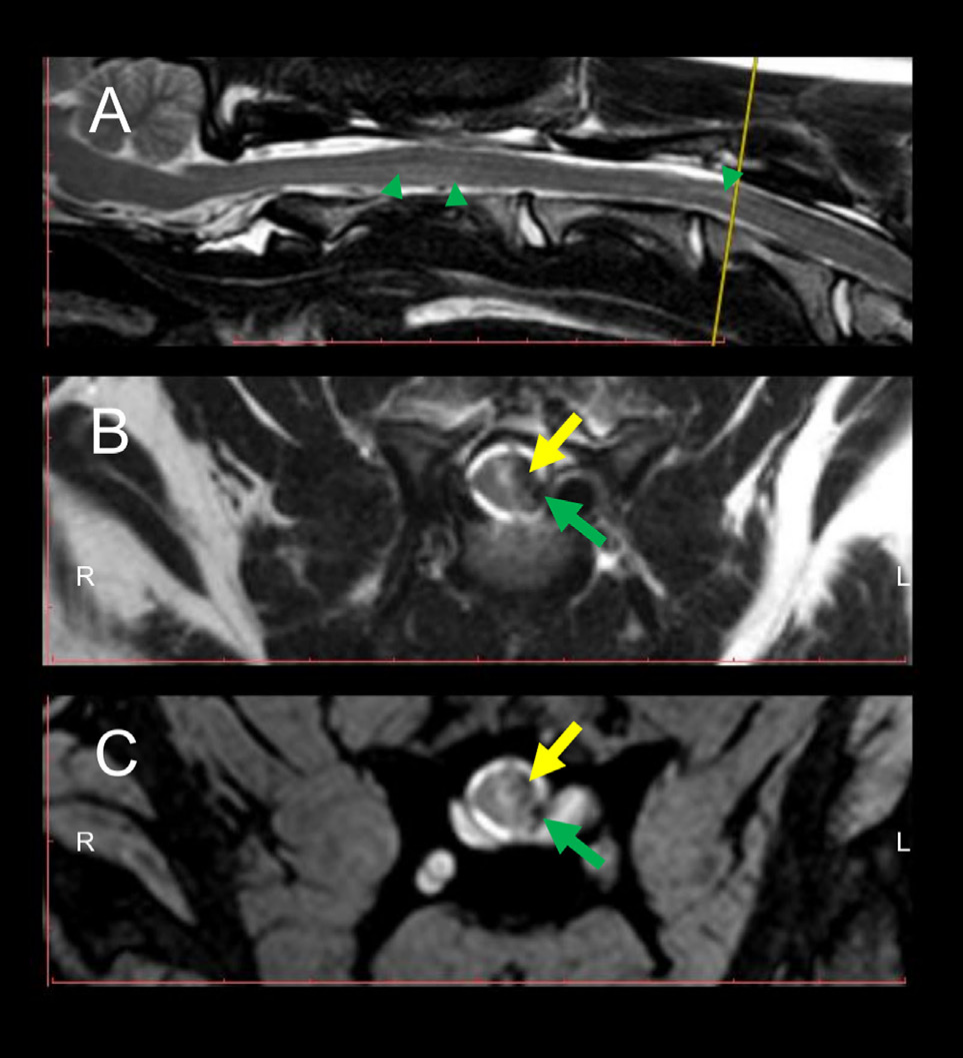
Follow-up MRI of the cervical spinal cord (SC) 8 weeks after initial presentation at the clinic. Intramedullary hyperintensities are present in the sagittal T2W sequence **(A)** dorsally to the vertebral body of C2 and at the level of C3–C4 (green arrowheads) in comparison to signal intensity of non-affected SC segments. Transversal T2W **(B)** and GRE **(C)** sequences depict intramedullary hyperintensities and concomitant presence of hypointense intramedullary lesions at the level of the left dorsal horn and left lateral white matter tracts (yellow arrows) as well as in the subarachnoid space (green arrows). The yellow line in the sagittal T2W sequence **(A)** indicates the level at which transversal sequences are depicted. Abbreviations: T2W, T2-weighted; GRE, T2*, gradient echo.

Eight weeks after initial presentation, follow-up general and neurological examinations, CBC and serum biochemistry, CSF analysis, and MRI of the cervical SC were performed. Same MRI protocols as used during the first examination were applied in the follow-up. At this time point, the dog was ambulatory showing a mild spastic tetraparesis, having the left limbs more affected and did not show any signs of pain during paravertebral palpation of the cervical region. Neuroanatomical localization during follow-up was consistent with a C1–C5 SC lesion. CBC and serum biochemistry were unremarkable and CSF taken from the cerebellomedullary cistern revealed a mild elevation of total protein content (value: 37.11 mg/dl; RI < 25 mg/dl). MRI revealed a focal intramedullary T2W hyperintensity at the level of C3–C4 with a presence of a left lateral T2W, GRE hypointense, and T1W hypo- to isointense structure, in comparison to unaffected SC segments, compatible with chronic hemorrhage, most probably residually lying within the subarachnoid space. Same signal pattern was noted within the SC parenchyma, suggesting focal intramedullary bleedings in the chronic stage (Figure 2). At this time point, subarachnoidal space dorsal and ventral to the SC in sagittal images appeared continuously T2W hyperintense. Additionally, a focal ventral intramedullary hyperintensity was noticed at the level of C2 (Figure 2; green arrowheads).

## Background

MRI represents a fundamental clinical tool for the understanding of the severity, extend, and localization of hemorrhages affecting the central nervous system (2). Temporal characterization of hemorrhage using MRI is challenging, as signal intensity in the different sequences varies depending on hemoglobin degradation product stages (3). Evolvement of signal intensity of canine blood has been recently described during *in vitro* and *in vivo* experimental studies (4, 5); however, the earliest time point in which signal intensity was recorded in such studies was 12 h after the hemorrhage was induced. Moreover, as hemorrhagic lesions are often associated with mass effect compressing the SC, further characterization of each temporal stage of bleedings may be fundamental for ruling out neoplastic and inflammatory processes and subsequently for clinical decision making (6).

MRI signal characteristics of early-stage hyperacute subarachnoid hemorrhage have been previously reported in humans (7). Nonetheless, An and colleagues (5) recently demonstrated different signal intensity patterns in the subacute stage of hemorrhages between canines and humans, suggesting that direct extrapolation of human data for interpretation of MRI blood signal intensity in dogs may not be completely accurate.

This case report describes the MRI findings of an early-stage hyperacute cervical intradural, extramedullary hemorrhage causing compression of the SC.

## Discussion

Subarachnoid bleedings within the vertebral canal represent an unusual condition in dogs and humans (6, 8, 9). Immune-mediated diseases causing vasculitis and consequently rupture of blood vessels within the subarachnoid space have been previously described in humans (2, 10, 11). Steroid-responsive meningitis-arteritis (SRMA) is a common inflammatory disease causing meningitis in young dogs (1). In SRMA, a necrotizing fibrinoid arteritis occurs within the leptomeninges (12). Moreover, neutrophilic leukocytosis with presence of left shift is a common finding in dogs with SRMA (1). Although coagulation time alterations are not commonly described in SRMA, increased prothrombine and partial thromboplastin time values could be affected during systemic inflammatory diseases and could be an early indicator of disseminated intravascular coagulation (13).

Albeit performing a CSF tap in a dog with subarachnoid hemorrhage due to vasculitis represents a risk that could lead to further bleeding within the leptomeninges, CSF analysis and characterization are considered to be pivotal for the diagnosis and subsequently prompt treatment initiation of dogs with SRMA (1, 12). Increased levels of IgA in both, serum and CSF, have been shown to be a sensitive diagnostic tool for the diagnosis of SRMA; however, around 10% of the patients may be classified as false negatives (14). In this case, elevation of IgA levels in CSF could be explained due to complete disruption of the blood–SC barrier causing free entrance of this protein into the subarachnoidal space.

Signal intensity within the core of the round-shaped lesion was increased in all performed sequences and suggests presence of peracute arterial bleeding, which is typically characterized by high concentration of oxyhemoglobin within the erythrocytes (3, 7). Oxyhemoglobin displays diamagnetic properties, contrary to later stages of hemoglobin degradation such as deoxy-, methemoglobin, or hemosiderin with paramagnetic behavior (7). Therefore, an absence of lesional signal voidance in GRE sequences, an otherwise characteristic sign for hemorrhage, is to be expected at this stage.

Moreover, additional to the round-shaped structure compressing the SC in the described dog, a later stage of hyperacute hemorrhage is suspected to be present and distributed along the subarachnoid space visualized dorsal and ventral to the SC in sagittal T2W images, as signal intensity is depicted mildly inhomogeneous and hypointense in T2W, T1W, and GRE sequences, probably due to an increase in deoxyhemoglobin at these localizations (7). Nevertheless, rupture of small venous vessels could cause flow of deoxyhemoglobin rich erythrocytes into the subarachnoid space as well. Due to the larger magnetic moment of impaired electrons, paramagnetic substances cause magnetic field inhomogeneities and subsequently susceptibility effects in GRE sequences (3). Additionally, strong contrast enhancement of the lesion in T1W sequences suggests still an active bleeding process or a continuously disrupted blood–SC barrier.

Interestingly, in sagittal and transversal T2W sequences, signal intensity within the structure compressing the SC varies in the dorso-ventral axis, showing a clearly defined division. This finding suggests a separation and sedimentation of the cellular components of blood during first stages of bleeding (3). Since the dog was positioned in dorsal recumbency for image acquisition, the segment showing lower signal intensity is seen dorsally and it represents cellular accumulation before clot retraction occurs; conversely, the supernatant, which is presumably mostly composed of blood plasma is the most T2W hyperintense portion of the lesion (3).

Surgical decompression of the SC is the gold standard for treatment of subarachnoid hemorrhages causing SC compression in humans; nevertheless, a complete recovery of neurological deficits is achieved in approximately 40% of the cases (15). In dogs, surgical treatment seems to have a positive impact in the outcome of dogs with extramedullary hematomas as well (6). Medical treatment with corticosteroids was preferred in this case, as an immune mediated vasculitis was the highly likely underlying disease and the coagulation profile of the patient suggested a high risk of intraoperative bleeding. Moreover, artificial ventilation during the first 24 h was chosen as a supportive measure due to peracute worsening of clinical signs before performing MRI scan and suspected active hemorrhage that could eventually further compress the SC.

In follow-up MRI, regression of the round-shaped structure compressing the SC was evidenced. Moreover, T2W and GRE hypointense areas located in the subarachnoidal space and intramedullary are compatible with chronic stage of hemorrhage (5). During this stage, remaining extracellular hemoglobin is oxidized to ferritin and hemosiderin, which is phagocytized by macrophages and accumulated within the lysosomes (3). Hemosiderin and ferritin are highly paramagnetic, causing therefore susceptibility effects in GRE sequences (3, 7).

As conclusion, early-stage hyperacute hemorrhages in dogs display similar MRI characteristics as in humans, where hyperintensities in T2W, GRE, SPAIR, and FLAIR sequences and strong contrast enhancement in T1W may be evidenced.

## Ethics Statement

As this manuscript does not describe any experimental procedure or clinical trial, but it represents a report of a single clinical case presented to a referral teaching veterinary hospital, no ethical approval was needed. The owner signed a consent form that the dog’s data can be used for scientific studies.

## Author Contributions

AW-L was involved in the clinical approach of the case, interpreted MRI images, and wrote the manuscript. E-IH was involved in the clinical approach of the case. KH was involved in the clinical approach of the case. AT supervised the clinical approach of the case, interpreted MRI images, and discussed and critically revised the manuscript. All authors participated in revising and correcting the final version of the manuscript.

## Conflict of Interest Statement

The authors declare that the research was conducted in the absence of any commercial or financial relationships that could be construed as a potential conflict of interest. The reviewer TP and handling editor declared their shared affiliation.
