# Supplementary material for: MRI Findings of Early-Stage Hyperacute Hemorrhage Causing Extramedullary Compression of the Cervical Spinal Cord in a Dog with Suspected Steroid-Responsive Meningitis-Arteritis
Source: Front Vet Sci. 2017 Sep 27;4:161. doi: 10.3389/fvets.2017.00161 (PMC5623665; doi:10.3389/fvets.2017.00161)
Supplement: Supplementary file 1 [file Table_1.DOCX]

**Supplementary Table 1.** Complete blood count, serum biochemistry values, and coagulation profile.

|  | Value | Reference intervals |
| --- | --- | --- |
| Leucocytes (x10^3^ cells/µl) | 35.12 | 6-12 |
| Lymphocytes (x10^3^ cells/µl) | 5.9 | 1-4 |
| Neutrophils (x10^3^ cells/µl) | 25.29 | 3-10 |
| Banded neutrophils (x10^3^ cells/µl) | 2.81 | 0-3 |
| Monocytes | 1.05 | 0-0.5 |
| Erythrocytes (x10^6^ cells/µl) | 6.34 | 6-9 |
| Hemoglobin (g/dl) | 14.7 | 15-19 |
| MCV (fl) | 69.1 | 60-77 |
| MCH (pg) | 23.2 | 17-23 |
| MCHC (g/dl) | 33.6 | 31-34 |
| Thrombocytes (x10^3^ cells/µl) | 229 | 150-500 |
|  |  |  |
| ALT (U/l) | 21 | <50 |
| GLDH (U/l) | 1.2 | <6 |
| ALP (U/l) | 307 | <250 |
| CK (U/l) | 92 | <90 |
| BUN (mg/dl) | 17 | 20-50 |
| Creatinine (mg/dl) | 0.3 | <1.4 |
| Lactate (mg/dl) | 41.5 | 4.5-22.5 |
|  |  |  |
| PTT (s) | 16 | 10-13.1 |
| PT (%) | 65 | 75 -130 |
| Von Willebrand Factor (%) | 153.7 | 50-180 |

MCV: mean corpuscular volume; MCH: mean corpuscular hemoglobin; MCHC: mean corpuscular hemoglobin concentration; ALT: alanine aminotransferase; GLDH: glutamate dehydrogenase; ALP: alkaline phosphatase; CK: creatine kinase; BUN: blood urea nitrogen; PTT: Partial thromboplastin time; PT: Prothrombin time.
